# Introductory Lectures

**Published:** 1849-01

**Authors:** 


Art. IV.—*Introductory Lectures. An Introductory Lecture* delivered in the Medical College of Georgia, at the opening of the annual session, Nov. 6th, 1848. By Joseph A. Eve, M. D., Prof. of Obstetrics and Diseases of Women and Infants.

*An Introductory Lecture on the Coinciding Tendencies of Medicines.* By Jared P. Kirtland, M. D., Prof. of Theory and Practice of Medicine, and Phyiscal Diagnosis in the Medical Department of the Western Reserve College, at Cleveland, Ohio.

*An Introductory Lecture* delivered to the class of Midwifery and Diseases of Women and Children, in Jefferson Medical College, Oct. 18th, 1848. By Charles D. Meigs, M. D,

The lecture of Dr. Eve treats of a variety of subjects, the most prominent of which is Medical education. He reviews, somewhat in detail, the various recommendations of the Medical convention in regard to preliminary education, the extension of the lecture-term, &c., and expresses a hearty concurrence in all the suggestions which look to the elevation of the profession. The tone of the lecture is frank, earnest, and manly. Its author does not hesitate to speak of abuses wherever he finds them; but while inveighing against them with a generous indignation, he never loses sight of the charities of the christian, nor of the language that befits the refined physician.

We fully agree with Dr. E. in the following sentiments:

It would certainly be bad policy to place any obstacle in the way of those who would attend lectures—the more generally they are attended the better, the more general the diffusion of medical knowledge among the people the better for the profession, as nothing would so effectually suppress empiricism. If any preparatory education be required, let it be demaded of the candidate when he applies for a degree; let him undergo an examination to test his qualifications; for little dependence can be placed on certificates, especially when the certifiers are remote and unknown. It is well known with what facility certificates can be obtained, by any person and for almost any purpose. The system of certificates would favor the unprincipled and bear hard on the more conscientious;—let all be subjected to the same ordeal. Instead of requiring certificates to prove extrinsic and unimportant points, as age, time of reading, &c., let the colleges adopt every method by which to improve their courses of instruction, to afford the pupil greater facilities, more extended opportunities of acquiring knowledge, and then raise the standard of qualifications proportionally higher. We believe these the true principles of reform, the only method by which a reformation can be accomplished.

The subject of Dr. Kirtland's lecture is the "*Coinciding Tendencies of Medicines*," an idea for which he acknowledges himself indebted to Drs. Miner and Tully, whose work on Fevers, published about twenty-five years since, many of our readers have doubtless seen. He regards the subject as one of exceeding interest, though students do not relish the doctrine, "that prudence, and not boldness, should be exercised in treating the sick." Some of the instances in which coinciding remedies may create factitious diseases, are referred to.

Thus, remedies will coincide if not adapted to the fulfilment of the indications; or if not adapted to the grade, or if not timed to the stage, or not proportioned in power to the amount, of the disease. They will coincide if employed under a false diagnosis, or if disproportioned in power to the amount of vitality remaining in the system. They will also coincide if not suitably qualified, or if their use be continued after they have accomplished certain changes in the system.

The following illustration of the coincidence resulting from a false diagnosis is given:

"A careless practitioner may mistake a fever and local disturbance, arising from worms, and teething, for peritoneal inflammation. He resorts to blood-letting and antimonials—they coincide; morbid irritability rages, and at length the brain sympathizes, and convulsions end the scene. Perhaps such a practitioner never considers how much he contributed to the result.

"Malaria, the cause of Fever and Ague, extends its influence over the west, and imparts peculiar tendencies to our diseases. Its effects are masked under the form of every ailment that occurs.

"In its irregularities, it more frequently manifests itself under the symptoms of a disordered state of the nervous system. One case may be neuralgia, another apoplexy, and a third, epilepsy, and so on through the list of nervous diseases. In one instance, it may appear as irritation, another inflammation, and another a compound of both.

A practitioner, not intimately acquainted with this sub ject, would, probably, at the first step make a false diagnosis, and before the expiration of two days of treatment, he would be surrounded by so many perplexities and anomalies, that he would be disposed to give up the case in despair."

The other propositions are illustrated by appropriate ex amples, and the subject, so well treated, ought, we think, to have interested the pupils of the learned professor. We have read his lecture with great pleasure.

The lecture of Dr. Meigs is marked by the author's peculiarities of style, but contains all his elevation and vigor of thought, and strikes us as an eloquent and beautiful production. Whatever else the reader might deny its author, he would be sure, on rising from a perusal of this lecture, to concede to him genius, that loftiest attribute of mind. We quote a few passages, and should hardly fear to do so at random, for every page gives proof of that rare faculty. Take for example the following, in which he is contrasting Philadelphia as it was,and as it has become under the sway of the scholar.

"It is scarcely more than one hundred and fifty years ago—and the men that saw it are but just lately dead—that the spot upon which we stand was a dark primeval forest, the haunt of the moose, the deer, the wolf, and the fox.

"Here the Lenni Lenape man crept with bow and hunting-spear nearer and nearer to the wild beast, whose spoils constituted his wealth, and satisfied his imperious necessities.

"The man had a soul that was filled, as Milton says, with 'vain imaginations, phantasms, and dreams, distempered discontented thoughts, vain hopes, vain aims, inordinate desires.' He was sunk in gross ignorance and vice, and the darkness of night was spread over his lot, and that of his race.

"Some human sympathies had he, mayhap, at the sight of his wife or his new-born child; and some sensual pleasure, in the curling incense of his tobacco smoke, as it wrapped his senses in its anæsthetic cloud. But a dark fiery passion *ruled* his life, and war and revenge were his *ideal*. The scalp-pole, with the gory emblems of his strength and courage, or the bloody corpse of his enemy, lying stark and silent, cold and gashed before him, procured for the Man emotions the most to be hoped for in his then present evil world. Such was the Philadelphian.

"But now, in this short space that we are thinking of, the bloody and cruel red man is clean gone out of our sight, and his record can scarce be found—the bramble and the thorn, the brake and tangled thicket, and the thousand-year-old oak, have given place to streets of palaces, the homes of a luxurious and refined civilization. The wild beast of the forest is no longer seen in his ancient lair, where the caparisoned horse bends his pliant willing neck to the rein; and the surly bear, and grim ferocious wolf, no longer gnash their teeth in paths where our little children pursue their toys in security. The lodge of the Indian is gone, and the marble hall is in its place. No more doth the scalp-pole lift its mournful trophy in the air, where the banner of the Republic throws out its stars and stripes to the winds that love to leap to the embrace of that beautiful and glorious emblem of our enlightened *liberty* and union, in which lie our strength and our happiness.

"Now is not this a marvellous change, to be effected, too, in so short a time! What was the necromancy that wrought it?

"Man was here then.

"Man is here now.

"What makes the difference—if we be, in truth, all alike, sons of the common Adam, father of men?

"What is it that hath swept away, as by a stroke of Merlin's wand, forest, and lodge, and wild beast, and wilder man, and planted in their stead a great and flourishing city, with its 380,000 Christian souls of its population,—abiding here in security and tranquillity, under wholesome and equal laws, the exponents of a true morality, and the guardians of it?

"We are here in the midst of plenty; we are surrounded with all the consolations of civilization. We have abundant markets; vast stores of corn, wine, and oil; streets cleansed, lighted and graded, and guarded; places of worship, magnificent libraries; public walks, and gardens and squares; hospitals and alms-houses for the sick and the wounded, the deaf and the blind, and the lame and the poor. Benevolent institutions rising up every day, and emulous to do whatever is possible for the amelioration of those who are ignorant, or vicious, or unfortunate; schools opened for all the children of the people, without fee or price; and to all this catalogue of good things here enjoyed—a confident belief that the same advantages are to be kept in store for our children, and our children's children, for generations that are as yet unborn.

"Here are gathered together the innumerable gifts of commerce; whatsoever the arts have produced of useful or agreeable, are here placed within the reach of those who desire them for use or for pleasure.

"Now what is it that hath wrought this great and miraculous change?

"All this is the work of the Scholar; for it is he who has led his fellow-man out of the grossness and ignorance and depravity of his natural state—that has taught him arts and letters, and converted him, from being a savage, into the more elevated condition of the Christain and gentleman. It is the Scholar that has broken asunder the manacles of the woman, and freed her and disenthralled her, from being the bond slave of the man, his beast of burthen and the mother of his child, to take her station as his coequal companion. The Scholar is man's Teacher and Guide. He it is who struck the scales from his eyes, and opened his deaf ears, and unsealed his closed senses, and called upon him with his voice, and showed him by his example, the way in which he should walk; invited him to spurn the degradation and bondage of ignorance and depravity, and to come up and be partaker with him of knowledge, which is freedom, and of virtue, which is happiness.

"It is the Scholar who hath done all this, and none else could have done it."

Take, again, the following—

"Who planted the sleep-giving poppy in the garden of the world? Who raised the tall and graceful cinchonas on the slopes of the Cordilleras? Who lifts up and supports and draws out the gracile bending stem of the one, or upholds the rigid trunk of the other? Who constructed the capsule and unfolded the tender blossom, and commanded the sanative or the pain-compelling juices to stream through the tubes and gather themselves together for the weary, sleepless, hopeless sons of the children of men? Was this an accident? or was it out of design, a remedy? Or are medicines appointed by the Author of the world, and you the ministers to employ and guide them? And did the *idea* of it exist before the root was buried in the ground? And you, young gentlemen, were you there too? Did that eye see you then as you sit before me now? Did He ordain the tympan and the semicircular canal that now hears my voice calling upon you, and appealing in vehement words, and a strong desire, that you should become indeed the missionary physician you were designed to be? Fulfil the precosmic idea of your nature and creation. Flee from every temptation that might draw you aside from the appointed possible track, and learn to know and to feel how honorable a thing it is to be a physician in deed and in truth—a messenger sent from above to earth, and by an absolute command ordained to the high vocation of the Scholar, and to the elevated ministry of the Iatrist."

We should suppose there is no reader who would not relish such poetry of thought and of language, even in an introductory lecture to a class on midwifery and the diseases of women and children.

